# Assessment of the Possibilities of Developing Effective Building Thermal Insulation Materials from Corrugated Textile Sheets

**DOI:** 10.3390/ma19010188

**Published:** 2026-01-04

**Authors:** Sigitas Vėjelis, Aliona Drozd, Virgilijus Skulskis, Saulius Vaitkus

**Affiliations:** 1 Building Materials Institute, Faculty of Civil Engineering, Vilnius Gediminas Technical University, Linkmenu Str. 28, LT-08217 Vilnius, Lithuania; 2 Institute of Economics and Rural Development, Lithuanian Centre for Social Sciences, A. Vivulskio Str. 4A-13, LT-03220 Vilnius, Lithuania; virgilijus.skulskis@ekvi.lt

**Keywords:** hemp fibres, corrugated textile sheets, corrugated thermal insulation, thermal conductivity, mechanical characteristics

## Abstract

Low-density thermal insulation materials tend to settle during operation or under small loads. Resistance to loads and settling is ensured by increasing the density of thermal insulation materials several times. This increases the weight of the material and the structure and production costs. In this work, using various technological processes, corrugated textile sheets and thermal insulation materials were produced from textile fabric. The development of such materials as effective thermal insulation materials for building insulation has not yet been studied. The corrugation of textile sheets enabled the thermal insulation material to exhibit good mechanical and deformation properties without increasing its density or thermal conductivity. The density of the specimens of the thermal insulation material made from corrugated sheets ranged from 76.8 to 51.9 kg/m^3^, and the thermal conductivity ranged from 0.0535 to 0.0385 W/(m·K). The most significant influences on density and thermal conductivity were the wave size and the adhesive layer. The density of unglued sheets of the same composition ranged from 51.3 to 29.8 kg/m^3^, and the thermal conductivity ranged from 0.0530 to 0.0371 W/(m·K). The highest compressive and bending strengths were observed in thermal insulation materials prepared from finely corrugated sheets. Their compressive stress at 10% deformation was 17.3 kPa, and their bending strength was −157 kPa. In comparison, the compressive stress of thermal insulation materials of the same density as non-corrugated sheets was only 0.686 kPa and, in the case of bending strength, 9.90 kPa. The obtained results show that the application of materials engineering principles allows us to improve the performance characteristics of materials.

## 1. Introduction

Thermal insulation materials for low-density buildings typically exhibit low strength and may undergo settlement during operation [1,2]. Low-density thermal insulation materials are developed to conserve raw materials and reduce costs [3]. Using methods known in mechanical engineering, materials can be strengthened by corrugation. The term ‘corrugated’ in general describes a series of parallel ridges and furrows. A structure with a corrugated surface formed by folding, moulding, or other manufacturing methods is called a corrugated structure [4]. Corrugated construction has been used to create a variety of structures for the construction industry. The most common types used in the construction sector are corrugated tubes [5], wood-based corrugated panels [6], metal structures for wall and roof coverings, and sandwich panels [7,8,9]. In general, the most widely known corrugated construction is corrugated paperboard [10,11,12]. Corrugated paperboard is produced by adding two face sheets (also known as liners) as upper and lower surfaces to the corrugated sheet (also known as core, medium, or fluting) [13]. By selecting appropriate shapes, dimensions, and materials for the face sheets and corrugated core, a range of stiffness and strength can be achieved while keeping the corrugated panel weight low. The structural characteristics of this corrugated structure depend mainly on the lightweight corrugated core, which separates the face sheets and provides the panel with the necessary stiffness.

Corrugated cardboard not only has good mechanical properties but can also be used to insulate heat and sound [14,15]. Heat and cold retention are significant when corrugated paper is used to transport hot or frozen products [16]. Corrugated paper products do not have widespread application as thermal insulation materials for several reasons: low water resistance [17,18], anisotropy [19], and limited thickness [20]. Low water resistance is associated with the raw materials used to produce corrugated paper, starch, and wood pulp [21,22,23]. Corrugated products are characterized by anisotropy due to the production method [24]; therefore, the products have good strength properties in one direction and poor properties in the other direction. The manufacturing process constrains the product’s thickness: the paper sheet is corrugated with toothed rollers and immediately glued to maintain its shape. In this way, 2–9 layers are combined, and the thickness of one layer is between 0.8 and 5 mm [25,26].

**Figure 1:**
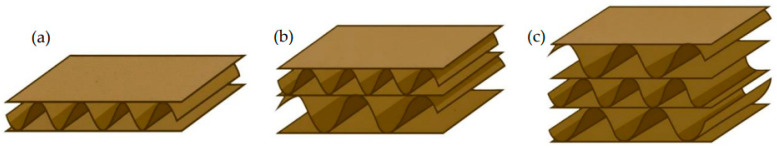
Types of corrugated board with different wave profiles and layer numbers: (**a**) single; (**b**) double; (**c**) triple.

The corrugated cardboard layouts of varying thickness and layer count are shown in Figure 1.

The basic geometric parameters that characterize corrugated boards are their thickness, wave height, pitch, and take-up ratio [27]. According to these parameters, the corrugated board flutes are divided into profiles [23]. The most commonly used profiles and their classification by parameters are presented in Table 1. In Table 1, the term “take-up ratio” refers to the ratio of the non-corrugated web length to the corrugated web length. Research by scientists [10,28] shows that the mechanical properties and strength of corrugated products depend directly on the flute profiles. Generally, larger flute profiles provide greater stability to the corrugated paperboard, while smaller flute profiles achieve better foldability and printability.

Another type of corrugated material is related to spacecraft. Corrugated thermal insulation materials, made from metal sheets, metalized sheets, or plastic films coated with aluminum, are used to insulate parts of spacecraft [29,30,31,32,33]. Metallized sheet insulation is produced by cross-laying, i.e., individual corrugated sheets are joined perpendicular to the corrugations, thereby imparting good strength characteristics in both the length and width directions [29]. Individual layers can be joined by welding, fusing, hanging, stitching, etc. [29,30].

In summary, the literature review shows that a relatively narrow range of raw materials is used to produce corrugated cardboard products. In addition, the geometric parameters of corrugated sheets are changed only to assess their strength characteristics, and their effect on thermal conductivity is not evaluated. Selecting appropriate parameters and technological processes for preparing corrugated sheets from fibrous mats to produce effective thermal insulation materials can reduce raw material use and improve their performance properties. Effective thermal insulation materials with increased resistance to load effects would be suitable for use in many building envelopes where small or short-term loads are applied and where lower deadweight structures are required.

This research aimed to create a thermal insulation material from renewable agricultural resources that retains heat well and is resistant to short-term loads and settlement. To achieve this goal, hemp fibre sheets were used to produce corrugated sheets, which were then glued together to create a new thermal insulation material. Corrugated sheets with different wave sizes were prepared to assess their effect on the thermal conductivity, bending strength, compressive strength, and density of the thermal insulation material.

## 2. Materials and Methods

### 2.1. Materials

Hemp fibre mats with 20% polylactide fibres were used to create the corrugated sheets. Hemp fibres and polylactide were selected as the only raw materials, ensuring that the product is made from renewable natural resources. Mats were manufactured by the nonwoven textile manufacturing company JSC Neaustima (Šiauliai, Lithuania). Hemp fibres were purchased from JSC Natūralus pluoštas (Kėdainiai district, Lithuania). The main characteristics of the fibres are presented in Table 2. A fibrous hemp mat was produced with a surface density of 75 g/m^2^ and a thickness of 10 mm.

The prepared corrugated sheets are glued together. Universal polyvinyl acetate (PVA) glue (Briko D3, Briko, Kaunas, Lithuania) was used to bond the corrugated sheets. The main properties of the glue are presented in Table 3.

### 2.2. Preparation of Corrugated Sheets and Thermal Insulation Materials

**Figure 2:**
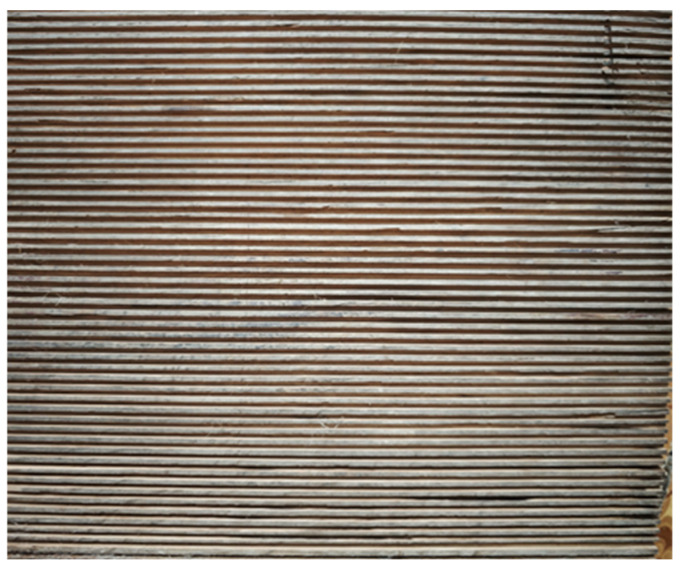
Form to produce corrugated sheets.

The resulting mat was used to make corrugated sheets. The sheets were produced by volume forming in a mould with grooves of different depths and widths. The moulds were made of hardwood by cutting out the grooves of the required size. A general view of the mould is shown in Figure 2.

The depths of the grooves of different shapes were 3, 5, and 7 mm to assess the effect of wave depth on the thermal and strength properties of the produced thermal insulation material. The groove pitch was 6, 10, and 14 mm, respectively.

**Figure 3:**
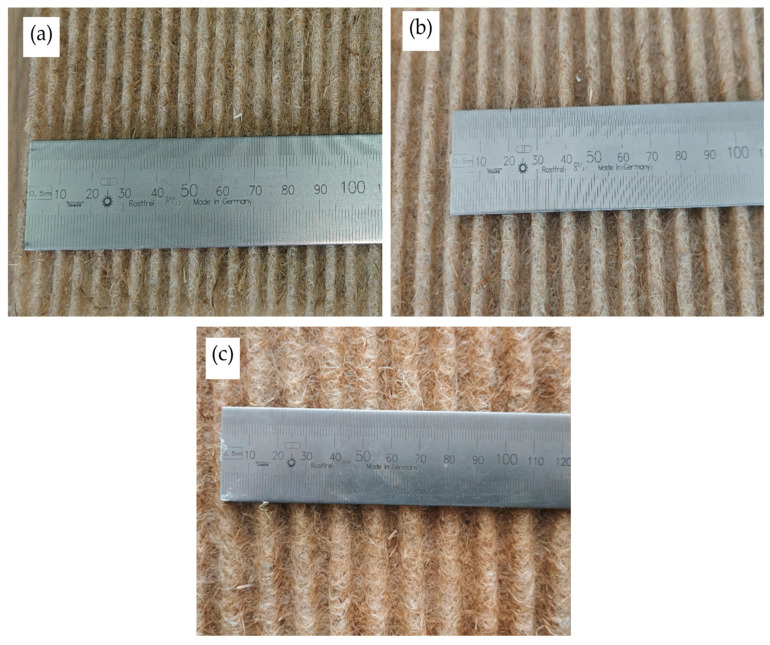
View of corrugated sheets made with wave height in mm: (**a**) 3; (**b**) 5; (**c**) 7.

The compressed mats in the mould were maintained in a controlled temperature chamber at 200 °C. After 12 min, the mould with the compressed mat was removed and left to cool for 60 min, allowing the binder to harden and maintain the corrugated sheet’s shape. The mat processing temperature and time were selected experimentally. When the mats were exposed to 200 °C for 12 min, polylactide melted uniformly throughout the entire mat volume, without compromising the strength indicators of the resulting corrugated sheet. At higher and lower temperatures, it was difficult to achieve uniform melting of polylactide and to optimize the mechanical properties of the resulting sheet by adjusting the curing temperature accordingly. The images of the corrugated sheets, obtained in various shapes, are shown in Figure 3.

**Figure 4:**
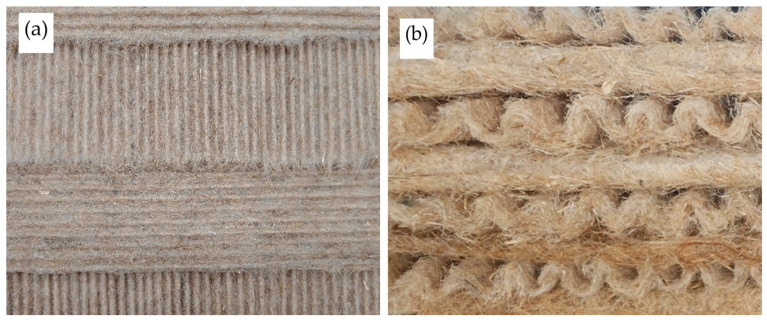
View of corrugated sheets for thermal conductivity measurement: (**a**) top view; (**b**) side view.

Once the required number of corrugated sheets is prepared, they are placed one on top of the other. Every second sheet is rotated 90° so that the waves of the different layers intersect perpendicularly. The sheets are then glued together with PVA adhesive. The sheets prepared for glueing are shown in Figure 4.

Depending on wave size, 8 to 18 layers are added in this way to obtain specimens of the desired thickness. Intermediate measurements, i.e., of unglued sheet specimens, were necessary to assess the adhesive’s influence on the density, thickness, and thermal conductivity of the thermal insulation material.

**Figure 5:**
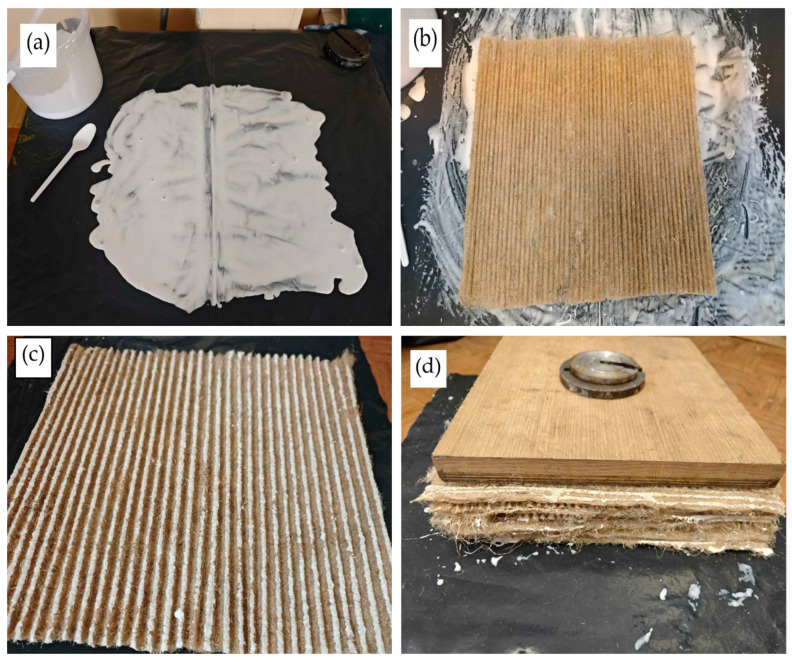
Installation of thermal insulation material from corrugated sheets: (**a**) specified thickness of the glue layer on the polyethylene film; (**b**) coating the tops of the waves with glue; (**c**) the tops of the waves are covered with glue; (**d**) the entire specimen is glued and pressed.

The corrugated surfaces of each sheet are immersed in pre-prepared glue. The thickness of the glue on the polyethylene film is maintained at 0.5–1.5 mm. A greater depth of the glue significantly increases the density of the prepared thermal insulation material and worsens its thermal conductivity. The glued sheets are pressed with a fixed load of 250 Pa. This load ensures sufficient sheet pressing so that the wave peaks of all sheets are connected to one another without deformation. The glued sheets are then left to dry naturally for 72 h, after which they are dried in a drying oven at 70 °C until a constant mass is reached. Typically, moisture is removed from the sample within 72 h, and a temperature of 70 °C is sufficient to induce the glass transition of PVA. The dried products are used to evaluate the performance properties. The glueing process for corrugated sheets is shown in Figure 5.

### 2.3. Test Methods

The density of the thermal insulation specimens was determined in accordance with EN ISO 29470 [35]. Specimens prepared for thermal conductivity were used for the tests. The load at which the thickness of the specimen was determined was 250 Pa.

Thermal conductivity was measured in accordance with EN 12667 [36]. For measurements, three specimens of thermal insulation material were prepared from each thickness of the corrugated sheet, with dimensions of 300 mm × 300 mm × 50 mm. Before testing, the specimens were conditioned in an environment at 50 ± 5% relative humidity and 23 ± 2 °C. The thermal conductivity values were determined at an average temperature of 10 °C.

The compressive strength was determined in accordance with EN ISO 29469 [37]. Five specimens measuring 100 mm × 100 mm × 50 mm were used for the tests. The test result was the compressive stress value at 10% deformation.

The bending strength was determined in accordance with EN 12089 [38]. Three specimens measuring 300 mm × 100 mm × 50 mm were used for the tests.

The fibre’s structure, the contact zones between them, and the macro- and microstructure of the specimens in the contact zones of the sheets were also evaluated. Scanning microscopy (SEM) was used for structural studies. A JEOL (Tokyo, Japan) scanning microscope was used for the studies.

### 2.4. Research Process Flowchart

**Figure 6:**
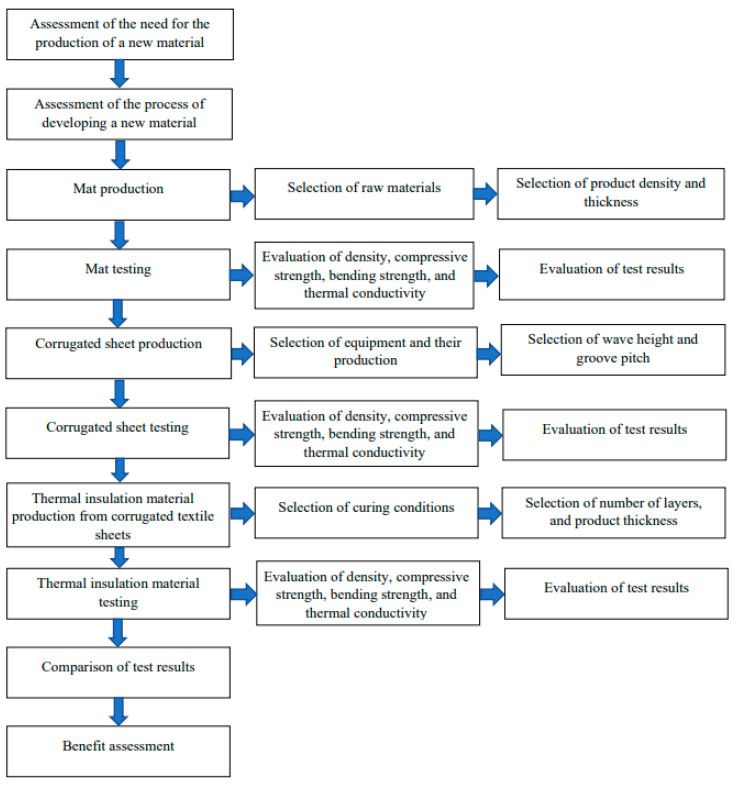
Research process flowchart.

Figure 6 presents the overall research process in flowchart format. The entire research process includes evaluating the development of new materials; evaluating the production processes for intermediates and new products; preparing for production; testing; evaluating and comparing results; and assessing benefits.

## 3. Results

First, specimens were prepared for thermal-conductivity testing. Nine groups of specimens were tested. Specimens from groups 1–3 were made of corrugated sheets of different sizes, arranged perpendicular to the direction of wave orientation (see Figure 4). Specimens from groups 4–6 were made of the specimens from groups 1–3, the sheets of which were interconnected with glue. The specimens from groups 7–9 were prepared from mats that were also used to produce corrugated sheets. Data on thermal conductivity specimens are presented in Table 4.

**Figure 7:**
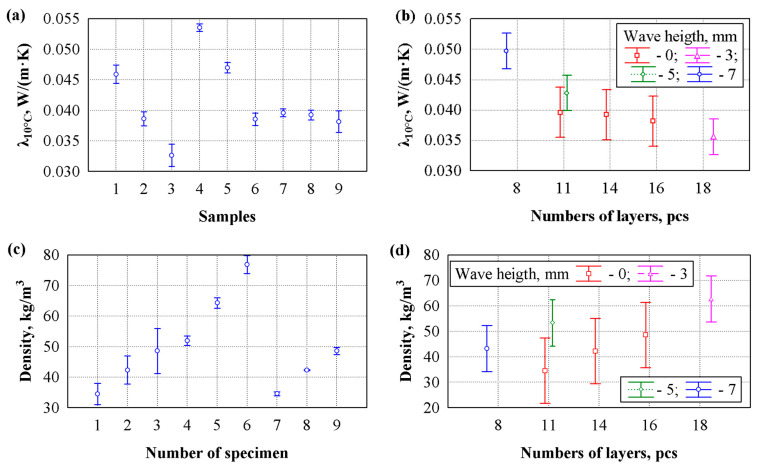
Analysis of experimental data on the thermal conductivity of specimens: (**a**) variation in the thermal conductivity by groups; (**b**) influence of the number of layers and wave height on the thermal conductivity; (**c**) variation in density by groups; (**d**) influence of the number of layers and wave height on the density; 1—coarse wave sheets; 2—medium wave sheets; 3—fine wave sheets; 4—coarse wave sheets (glued); 5—medium wave sheets (glued); 6—fine wave sheets (glued); 7, 8, and 9—non-corrugated mats.

Such groups of specimens enabled us to evaluate the effects of both wave size and the adhesive layer on the specimens’ density and thermal conductivity. The thermal conductivity and density results for the specimens are presented in Figure 7.

The densities of the specimens from groups 1 and 7, 2 and 8, and 3 and 9 were set equal to evaluate the influence of sheet corrugation on thermal conductivity. The specimens of groups 4, 5, and 6 were formed from the specimens of groups 1, 2, and 3, respectively, to evaluate the influence of adhesives on the density of the specimens. The analysis of the results showed that the density of fine corrugated sheets was almost 41% higher than that of coarse corrugated sheets and nearly 15% higher than that of medium corrugated sheets.

The bonding process significantly affected the specimens’ density. Adhesive layers between the corrugated sheets increased the density of specimens made from fine corrugated sheets by 58.0%, medium corrugated sheets by 52.0%, and coarse corrugated sheets by 50.4%. This indicates that a greater number of fine corrugated sheets results in more contact areas, thereby increasing specimen density.

The initial assessment of the thermal conductivity test results showed that, in all cases, the lowest thermal conductivity was observed in the specimens with the highest density in each group. It is known that in low-density materials, heat transfer occurs intensively through air [39,40]. In our case, heat is transferred through the air gaps between the fibres or between the waves. Increasing specimen density reduces the air gaps between fibres, and decreasing the wave size in corrugated sheets creates smaller air gaps between waves. Comparing specimens 1–3 and 4–6 shows that the contact zones of the adhesives significantly increase the thermal conductivity of the specimens formed from sheets with large waves. Thus, the increase is 35.0%. As the wave size of the specimen sheets decreases, the influence of the adhesives decreases. The thermal conductivity coefficient of the specimens formed from sheets with medium-sized waves increases by 23.0% due to the adhesive contact zones. In comparison, the thermal increase in the specimens with small waves is only 3.8%. Given the densities of the unglued and glued specimens, although the most significant density increase among specimens prepared from sheets with small waves was 41.0%, the effect on thermal conductivity was minimal.

An analysis of variance was performed on the thermal conductivity coefficient results shown in Figure 7a across the specimen groups. The *F*-criterion was 487, and *p* = 0, indicating a statistically significant difference in the thermal conductivity coefficient. The data from the observed specimen indicated a low risk of error at *p* < α, where α is the significance level, and 0.05 is the threshold for statistical analysis in the study. The *F*-criterion was used to determine the impact of various factors on the target criteria. If the *F*-criterion is sufficiently high, it would indicate a significant statistical effect. Therefore, the corresponding *p*-value will be small, indicating strong evidence against the null hypothesis [41]. After repeated analysis between individual groups, it was found that their mean values did not differ: groups 1 and 5—*F* = 6.92, *p* = 0.06 (0.0464 ± 0.000734 W/(m·K)); groups 2, 6, and 9—*F* = 0.654, *p* = 0.06 (0.0384 ± 0.000517 W/(m·K)); groups 7 and 8—*F* = 2.33, *p* = 0.20 (0.0394 ± 0.000331 W/(m·K)). The highest thermal conductivity coefficient is observed in specimens of group 4—0.0535 ± 0.000252 W/(m·K), and the lowest in group 3—0.0326 ± 0.000737 W/(m·K).

Figure 7c shows the variation in specimen densities used for thermal-conductivity testing, grouped by material. Analysis of variance of the specimen densities showed that the group means differed significantly (Figure 7c). The statistic of the *F*-criterion was 306, and *p* = 0. This indicates a statistically significant difference in the thermal conductivity densities of the specimens. After repeated analysis between individual groups, it was determined that the mean values of the following groups do not differ: groups 1 and 7—*F* = 0.0016, *p* = 0.97 (34.5 ± 0.900 kg/m^3^); groups 2 and 8—*F* = 0.0038, *p* = 0.95 (42.3 ± 1.178 kg/m^3^); groups 3 and 9—*F* = 0, *p* = 1.0 (48.6 ± 1.90 kg/m^3^). The highest density, 76.8 ± 1.19 kg/m^3^, is observed in the specimens from group 6. After repeated analysis, the relationship between thermal conductivity and specimen density was determined. It was found that density in this case does not have a decisive influence on the thermal conductivity coefficient. The following statistical values were obtained: R = 0.130; R^2^ = 0.0169; *F* = 0.206; *p* = 0.82. The correlation coefficient R measures the strength and direction of the linear relationship between two quantitative variables. The closer R is to 1 (−1 or 1), the stronger the relationship. The coefficient of determination R^2^ is a measure of the goodness of fit of a model. The coefficient of determination R^2^, evaluated within the range of 0 ≤ R^2^ ≤ 1, or expressed as a percentage [41]. Thus, the geometric structure plays a more critical role in heat-transfer behaviour than bulk density alone.

Next, an attempt was made to assess the influence of the thermal conductivity coefficient on the number of layers and wave height (Figure 7c). It was determined that the number of layers and wave height influence the thermal conductivity coefficient, and the following statistical values were obtained: R = 0.856; R^2^ = 0.733; *F* = 11.56; *p* = 0.00002. The highest thermal conductivity coefficient was observed in specimens with eight layers and a 7 mm high wave, with an average value of 0.0497 ± 0.00420 W/(m·K). The lowest thermal conductivity coefficient was observed in specimens with 18 layers and a three mm high wave, with an average value of 0.0356 ± 0.00328 W/(m·K).

In addition, an attempt was made to evaluate the influence of specimen density on the number of layers and wave height (Figure 7d). It was determined that specimen density affects both the number of layers and wave height. The following statistics were obtained: R = 0.691, R^2^ = 0.477, F = 3.84, *p* = 0.013. The highest density was observed in specimens with 18 layers and a three mm-high wave, with an average value of 62.7 ± 15.6 kg/m^3^. The lowest density is observed in specimens with 11 layers and a wave height of 0 mm (non-corrugated mats), with an average value of 34.5 ± 0.252 kg/m^3^.

**Figure 8:**
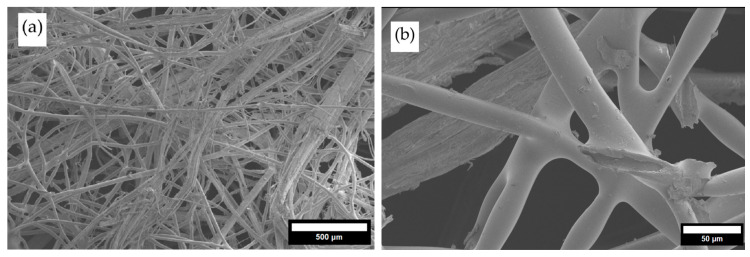
Structure analysis of corrugated thermal insulation: (**a**) general view of the fibres; (**b**) view of contact zones between fibres.

To explain the changes in thermal conductivity and density of the glued and unglued specimens, a structural analysis was performed. During structural analysis, the corrugated sheet’s structure, wave sizes and shapes, sheet thickness, wave contact zones, and sheet glue were evaluated. The sheet-structure study revealed numerous contact zones ensuring the required strength of the corrugated sheets (see Figure 8a,b).

**Figure 9:**
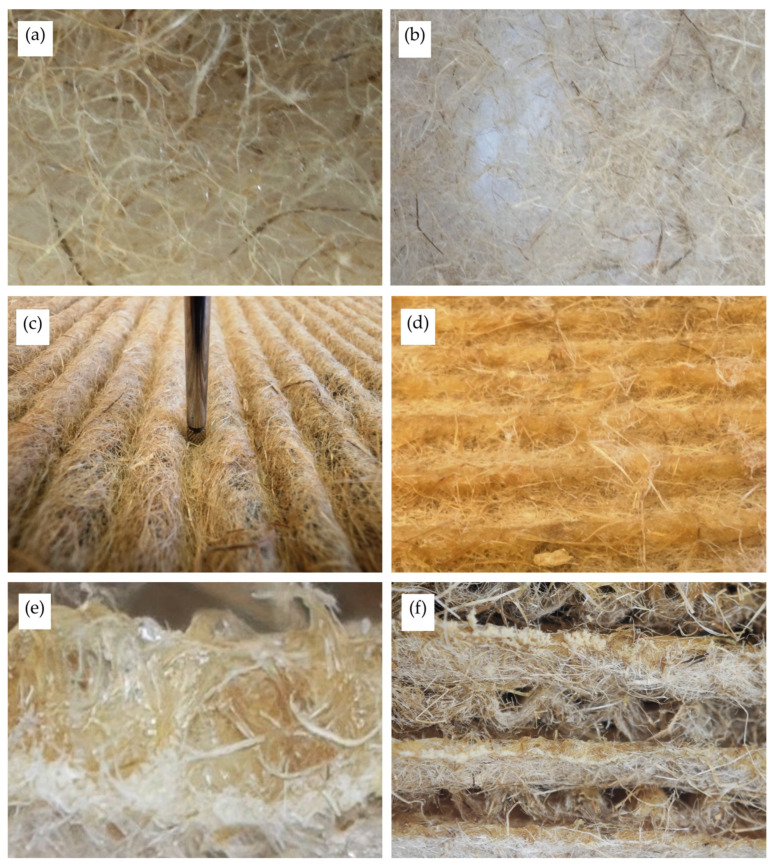
Structure analysis of corrugated thermal insulation: (**a**,**b**) arrangement of fibres in different areas; (**c**) thickness evaluation of unglued corrugated sheet; (**d**) thickness evaluation of glued corrugated sheet; (**e**) formation of a glue layer at the crests of waves; (**f**) glue layer between fibres.

In the resulting sheet, a relatively high heterogeneity in fibre distribution is observed, which is why both the gaps between fibres and the mat density vary (see Figure 9a,b).

An analysis of the thickness of unglued and glued sheets (see Figure 9c,d) was performed to assess the influence of the adhesive on sheet thickness changes. Due to the heterogeneity of the textile layers and the sheet-production method, the sheet thickness, regardless of the specimen’s wave size, varied from 0.26 to 0.61 mm at the wave crests. The thickness of the adhesive-coated sheet at the wave crests increased several times, ranging from 1.64 to 1.95 mm.

Analysis of the cross-sectional images of the glued specimens enabled us to assess the distribution of the adhesive layer within the specimens (see Figure 9e,f). Figure 9e shows that a continuous adhesive layer forms along the sheet’s wavelength. Based on the estimated wave thicknesses of the unglued and glued sheets (Figure 9e,f), the adhesive layer thickness ranges from 1 to 1.5 mm. Figure 9e shows that the adhesive layer is not homogeneous, with pores and gaps observed. Such porosity in the hardened adhesive could have had a minor impact on the overall increase in the specimen’s thermal conductivity. Furthermore, a minor increase in the thermal conductivity coefficient was observed because the strips were arranged perpendicular to the direction of heat flow. Hence, the increased thermal conductivity due to heat transfer through the solid material framework was minimal. It should also be noted that the cured adhesive bands of thermal insulation materials prepared from corrugated textile sheets with the smallest waves are narrower and range from 1.14 to 2.32 mm, and those with the most significant waves range from 1.96 to 3.28 mm. The difference is 51.4% on average and 23.7% when comparing specimens with small and medium waves.

**Figure 10:**
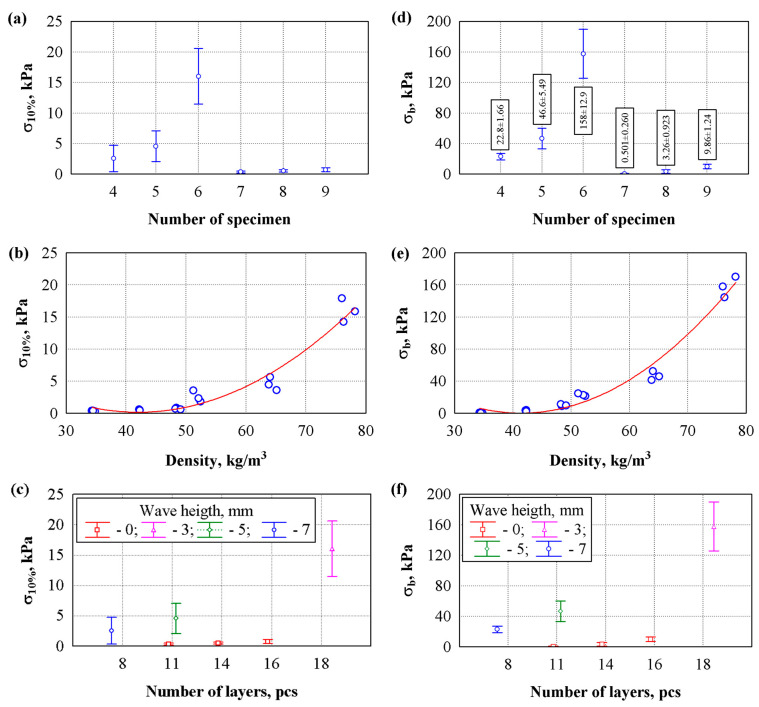
Studies of the mechanical properties of fibres composites: (**a**) variation in compressive stress by groups; (**b**) dependence of compressive stress on density; (**c**) influence of compressive stress on the number of layers and wave height; (**d**) variation in bending stress by groups; (**e**) dependence of bending stress on density; (**f**) influence of bending stress on the number of layers and wave height; ○: experimental data; ▬: experimental curve.

Figure 10 presents the results of compressive stress tests at 10% deformation. Specimens from groups 1–3 were prepared from non-corrugated mats, and their density was matched to that of thermal insulation materials prepared from unglued textile sheets with corrugations of different sizes. Specimens from groups 4–6 were prepared from glued textile sheets with corrugations of different sizes. Analysis of the test results indicates that the compressive stress values for specimens in groups 1–3 are very low, not exceeding 1 kPa. Meanwhile, the compressive stress of the specimens made from the same amount of fibrous raw material, but using corrugation technology and additional bonding of the contact zones, is significantly higher—from 7.3 times when the thermal insulation material specimens were prepared from textile sheets with the largest corrugations to 22.2 times when the thermal insulation material specimens were prepared from textile sheets with the smallest corrugations.

Figure 10a presents the variation in compressive stress by groups. The analysis of variance (Figure 10a) showed that the group means differed significantly (*p* < 0.05). The *F*-criterion statistic was 127 (*p* = 0). This indicates a statistically significant difference in the compressive stress results. Specimens from group 6 exhibit the highest compressive stress (16.0 ± 1.84 kPa), whereas those from group 7 exhibit the lowest (0.347 ± 0.0586 kPa). After performing an analysis of variance regression, it was determined that density affects compressive stress, and the following statistical values were obtained: *F* = 122; *p* = 0; R = 0.970; R^2^ = 0.942; S_r_ = 1.47 kPa. In addition, compressive stress as a function of density can be described by the equation (Figure 10b):

(1) $$\sigma_{10\%}=22.178-1.0476\cdot \rho +0.012462\cdot \rho^{2}$$

In addition, an attempt was made to assess the influence of compressive stress on the number of layers and the height of the wave (Figure 10c). It was determined that the number of layers and wave height influence the compressive stress; the following statistical values were obtained: R = 0.991; R^2^ = 0.981; *F* = 127; *p* = 0. We observe the lowest compressive stress in specimens with 11,14 and 16 layers and a wave height of 0 mm. The highest compressive stress is observed in specimens with 18 layers and a wave height of 3 mm.

Figure 10d shows the variation in the bending stress by groups. Analysis of variance (Figure 10d) showed that the group averages differ significantly, *F* = 321, *p* = 0. The highest bending stress is observed in the specimens of group 6—158 ± 12.9 kPa, and the lowest in group 7—0.510 ± 0.260 kPa. Furthermore, a variance-regression analysis revealed that density is associated with bending stress. The following statistical values were obtained: *F* = 245; *p* = 0; R = 0.985; R^2^ = 0.970; S_r_ = 10.4 kPa. The dependence of the bending stress on the density can be described by the equation (Figure 10e):

(2) $$\sigma_{t}=206.293-9.9549\cdot \rho +0.120217\cdot \rho^{2}$$

Figure 10f evaluates the influence of bending stress on the number of layers and the wave height. It was found that the number of layers and wave height affect the bending stress, the following statistical values were obtained: R = 0.996; R^2^ = 0.993; *F* = 320; *p* = 0. We observe the lowest bending stress in specimens with 11, 14 and 16 layers and a wave height of 0 mm. The highest bending stress is observed in specimens with 18 layers and a wave height of 3 mm.

Figure 10f presents the results of the bending strength tests. The results of the bending strength tests exhibit trends similar to those observed in the compressive stress tests. Analysis of the test results shows that the bending strength values of specimens from groups 1–3 are very low but change significantly even with a slight increase in density. When the density increases from 34.8 kg/m^3^ to 48.4 kg/m^3^, the bending strength increases from 0.501 kPa to 9.86 kPa, which is approximately 19.7 times or more than 1.4 kPa per 1 kg of specimen. Meanwhile, the bending strength of the specimens made from the same amount of fibrous raw material, but using corrugation technology and additional bonding of the contact zones, increases from 45.5 times when the thermal insulation material specimens are prepared from textile sheets with the largest corrugations to 16.4 times when the thermal insulation material specimens are prepared from textile sheets with the smallest corrugations.

## 4. Discussions

Corrugated materials are usually made of paper, but the production of such materials, as our research shows, can also be performed from textile sheets. Although paperboard is the best-known corrugated material, there is no exact data on how many times the strength indicators of paper increase after corrugation. This is determined by multiple factors, including the number of layers [27], the sizes and shapes of waves and their combinations [28,42], the efficiency of the adhesive layer [43], the thickness of the sheet [28], and others. When developing thermal insulation materials from textile sheets, these indicators must be evaluated, as it is essential to obtain a material with not only good strength parameters but also low thermal conductivity. Furthermore, agriculture has significant potential to provide a variety of fibres for diverse applications [44,45,46], including thermal insulation materials [45,46,47,48,49,50].

In this work, a new type of effective thermal insulation material is presented. It is unique in several respects: created using plant-based fibres and employing a new corrugation technology with heat treatment, the resulting materials exhibit several times higher strength indicators than non-corrugated materials of the same composition.

Although textile fibres are well studied and used in various fields, their use in producing thermal insulation materials from corrugated sheets has not yet been investigated. This is primarily related to technological parameters, as new production equipment is required. Corrugated paper is produced by folding waves of the desired size and shape with toothed rollers and immediately fixing them to the paper sheet with glue so that the waves do not lose their shape [51]. When producing a corrugated sheet from textile fibres, heat treatment is required to ensure that the resulting waves retain their shape after cooling and achieve relatively high strength. This is achieved by using thermoplastic fibres, which soften at high temperatures, allow the desired shape to be formed, and harden and retain that shape upon cooling.

Thermal insulation materials made from textile fibres are often used in the clothing industry. The thermal conductivity coefficient for these materials varies from 0.0307 to 0.0463 W/(m·K) [52]. Thermal conductivity is relevant for such products in ensuring thermal comfort. Meanwhile, strength indicators are usually crucial in the opposite direction from those for building thermal insulation materials; they must be soft and flexible to ensure sensory comfort, e.g., in winter jackets.

Natural fibres are also used as thermal insulation materials for building envelopes. The density of such products is usually low because natural fibres are significantly more expensive than mineral fibres. Typically, the thermal conductivity of natural fibre-based thermal insulation materials ranges from 0.0330 to 0.0750 W/(m·K), and the compressive and bending strengths are not reported [53,54].

Our new material still requires substantial research. It is essential to evaluate the durability of such materials, their moisture resistance, particularly in contact areas, and their long-term load-bearing capacity so that these materials can be used in building envelopes without altering their operational properties. It is also essential to evaluate the flammability of such a material, as it is likely to increase due to the numerous cavities. Due to their uneven surface, such materials should be particularly suitable for plastered structures. In this way, the thermal insulation material would be perfectly protected from possible flame effects. Also, good flame protection can be ensured by using it in production of sandwich panels.

A more detailed analysis of the composition of the mat’s raw materials, technological production factors, the influence of wave sizes and shapes, and the installation of the connecting layer of corrugated sheets will likely enable a significant reduction in the thermal conductivity coefficient and further improvements in strength indicators.

## 5. Conclusions

This research substantiates the feasibility of creating thermal insulation materials from corrugated textile sheets made from renewable agricultural resources.

Thermal insulation materials formed from sheets with varying wave sizes exhibit different operational properties. Specimens prepared from sheets with 3 mm-high waves had the lowest thermal conductivity (0.0385 W/(m·K)) and the highest compressive stress (16 kPa). Their thermal conductivity is comparable to that of classical effective thermal insulation materials, and their strength is 22.2 times that of specimens prepared from non-corrugated mats.

The formation of waves in the sheets has the most significant influence on the specimens’ bending strength. The bending strength of the specimens made from the same amount of fibrous raw material, but using corrugation technology and additional bonding of the contact zones, increases from 45.5 times when the thermal insulation material specimens were prepared from textile sheets with 7 mm high corrugations and up to 16.4 times when the thermal insulation material specimens were prepared from textile sheets with 3 mm high corrugations.

This work represents the first attempt to develop an effective thermal insulation material from corrugated textile sheets made from plant-based materials. To improve the properties of this new thermal insulation material, a series of experiments is required, primarily including the analysis of the composition of the mat preparation mixtures, the assessment of the influence of the density and thickness of the corrugated sheets, the methods of joining the corrugated sheets, and the evaluation of the effect of different wave heights and shapes in one sample.

## Figures and Tables

**Table 1:** Classification of corrugated cardboard profiles.

Profile	Take-Up Ratio	Height, mm	Pitch, mm
O	1.14	0.3	1.2
N	1.11–1.8	0.4–0.5	1.8
G	1.17	0.5	1.8
F	1.19–1.28	0.7–0.8	2.4–2.5
E	1.20–1.35	1.1–1.4	3.2–3.7
B	1.26–1.48	2.3–2.8	6.1–6.6
C	1.36–1.56	3.4–4.0	7.4–8.3
A	1.37–1.53	4.1–4.7	8.7–9.5
K	1.50	5.94	11.7
D	1.48	7.38	15.0

**Table 2:** Main parameters of fibres.

Type of Fibre	Length of Fibres, mm	Fibre Diameter, µm	Linear Density, dtex	Melting Point, °C	Colour	Producer
Hemp fibres	40–60	17–22	-	-	light brown	JSC Natūralus Pluoštas, Kėdainiai, Lithuania
Polylactide	51	16–19	4.4	130	white	Max Model S.A.S, Lyon, France

**Table 3:** Main characteristics of glue.

Glue Type	Colour	Moisture Resistance Class [34]	Open Holding Time, min	Compression Time, min	Viscosity, mPas
Universal	White	D3	5–15	20–40	11,000

**Table 4:** Corrugated sheets and mats data.

Group of Specimens	Form of Specimen	Number of Layers, pcs	Wave Height, mm	Thickness, mm	Density, kg/m^3^	Thermal Conductivity, W/(m·K)
1	Coarse wave sheets	8	7.13 ± 0.83	54.2 ± 0.31	34.5 ± 1.40	0.0459 ± 0.00061
2	Medium wave sheets	11	5.08 ± 0.47	53.3 ± 0.57	42.3 ± 1.86	0.0386 ± 0.00046
3	Fine wave sheets	18	3.02 ± 0.68	53.5 ± 0.76	48.6 ± 2.97	0.0326 ± 0.00074
4	Coarse wave sheets (glued)	8	7	54.3 ± 0.50	51.9 ± 0.62	0.0535 ± 0.00025
5	Medium wave sheets (glued)	11	5	51.0 ± 0.47	64.3 ± 0.70	0.0470 ± 0.00035
6	Fine wave sheets (glued)	18	3	52.3 ± 0.90	76.8 ± 1.19	0.0385 ± 0.00040
7	Non-corrugated mat	11	0	53.1 ± 0.36	34.5 ± 0.25	0.0396 ± 0.00027
8	Non-corrugated mat	14	0	55.2 ± 0.60	42.3 ± 0.06	0.0392 ± 0.00032
9	Non-corrugated mat	16	0	53.5 ± 0.36	48.6 ± 0.47	0.0381 ± 0.00071

## Data Availability

The original contributions presented in this study are included in the article. Further inquiries can be directed to the corresponding author.
